# Clinical Measurement of the Tibio-femoral Angle in Malay Children

**DOI:** 10.5704/MOJ.1507.005

**Published:** 2015-07

**Authors:** MI Mohd-Karim, AR Sulaiman, I Munajat, AH Syurahbil

**Affiliations:** Department Of Orthopaedics, Universiti Sains Malaysia, Kubang Kerian, Malaysia

**Keywords:** Tibiofemoral angle, children, maximum valgus, age

## Abstract

Background: This study was conducted to find out the age when tibiofemoral angle starts to be in valgus and reaches maximum angle. The differences of the angles between genders were also studied.

Methodology: This cross sectional study on tibiofemoral angle was conducted among 160 normal healthy children using clinical measurement method. The children between 2 18 months to 6 years old were assigned to 5 specific age groups of 32 children with equal sex distribution.

Result: This study had shown a good inter-observer reliability of tibiofemoral angle measurement with intraclass correlation coefficient (ICC) of 0.87 with narrow margin of 95% confident interval (95% CI: 0.73, 0.94). The mean tibiofemoral angle for children at 2 , 3 , 4 , 5 and 6 years old were 2.25° (SD=0.53), 8.73° (SD=0.95), 7.53° (SD=1.40), 7.27° (SD=1.14) and 6.72° (SD=0.98) respectively. The age when they achieved maximum valgus tibiofemoral angle was 3 years old. The maximum mean (SD) tibiofemoral angle for boys, girls and all children were 8.91° (SD=1.17), 8.56° (SD=0.62) and 8.73° (SD=0.95)respectively. The mean tibiofemoral angle showed no statistically significant difference between girls and boys except for the 5-year-old group, in which the mean TF angle for girls was 7.560 (SD=0.95) and for the boys was 6.970 (SD=1.26) with p-value of 0.037.

Conclusion: Measurement of tibiofemoral angle using the clinical method had a very good inter-observer reliability. The tibiofemoral angle in Malay population was valgus since the age of 2 years with maximum angle of 8.730 (SD=0.95) achieved at the age of 3 years.

## Introduction

The normal development of tibiofemoral angle evolves through a number of stages in a normal growing child. Salenius and Vankka^1^ found that the tibiofemoral angle was in varus position before one year and become neutral at the age of one year and 6 months. Then the knee angles become valgus at 2 to 3 years with maximum mean valgus of 12^0^ at 3 years old. The valgus angle of knee decreased to between 5^0^ and 6^0^ in children between 7 and 12 years. However, there have been contradicting data of normal ranges of knee angle in relation to age, transition time from varus to valgus and the maximum peak valgus of different populations and ethnic groups^1,2,3,4,5,6^.

The aim of this study was to test the reliability of clinical method for measurement of tibiofemoral angle. We investigated the pattern of tibiofemoral angle. among Malay children using this clinical measurement.

## Materials and Methods

This cross-sectional study was conducted from January 2011 till January 2012 in Kelantan, Malaysia. Children age ranged from 2 to 6 years were put into 5 specific-age-groups consisting of 32 children with equal sex distribution. The chronological age of the subject was rounded off to the nearest integer. For example, children from the age of 1 years and 7 months to the age of 2 years and 6 months were included in the age group of 2 years. All the children must be with the weight and height within 2nd and 98th centile. Children with skeletal and neuromuscular disorder and Limb Length Discrepancy more than 2 cm were excluded from the study.

The sample size was determined by using independent t-test calculation method in PS Software (Dupont and Plummer, 1997). Parameters that had been used were :α (Type 1 error) = 0.05; power (probability of correctly rejecting the null hypothesis) = 0.8. ; m (ratio of control to experimental patients) = 1. Based on the previous study done by Saini *et al*^4^, δ (a difference in population means) = 1.76, σ (is the within group standard deviation) = 2.2, the sample size required was 160 children after considering 20% dropout rate.

Tibiofemoral angle was measured according to the method described by Arazi *et al*^3^. Angle between the two lines was measured as tibiofemoral angle using goniometer while the children in standing position with patella pointing forward and either both knees or both ankles were just touching each other. The knees and hips joints were positioned in full extension with arm placed behind the back to increase the stability of posture. The first line was the line transacting anterior superior iliac spine and the mid-point of patella (mid-point of maximum mediolateral width measured by tape measure). The second line was the line transacting midpoint of ankle (midpoint between the medial mallelolus and lateral malleolus midpoint of patella. The tibiofemoral angle was positive for varus knee alignment and negative for valgus knee alignment. Reliability of the measurement by two observers was tested on 5 children of each age group using similar measurement material and technique (total of 25 children). Measurements on other children were done by a single person at two different intervals for each child and mean of two measurements was taken as final tibiofemoral angle.

All data entry and analysis was performed using the Social Science and Statistical Package (SPSS) version 20.0 which are licensed to Universiti Sains Malaysia, Kubang Kerian Kelantan. The means, standard deviations, and 95% confidence intervals (95% CI) were calculated for each age and gender group. The mean tibiofemoral angle for both boys and girls were tested using the independent t-test. Inter-rater reliability of tibiofemoral angle measurement was measured using with 95% confidence intervals to gauge the precisions of the ICCs.

This study was approved by Ethical Committee, School of Medical Sciences, Universiti Sains Malaysia, Kubang Kerian, Kelantan.

## Results

Between two observers was 0.87 indicating a very good reliability.

**Fig. 1:**
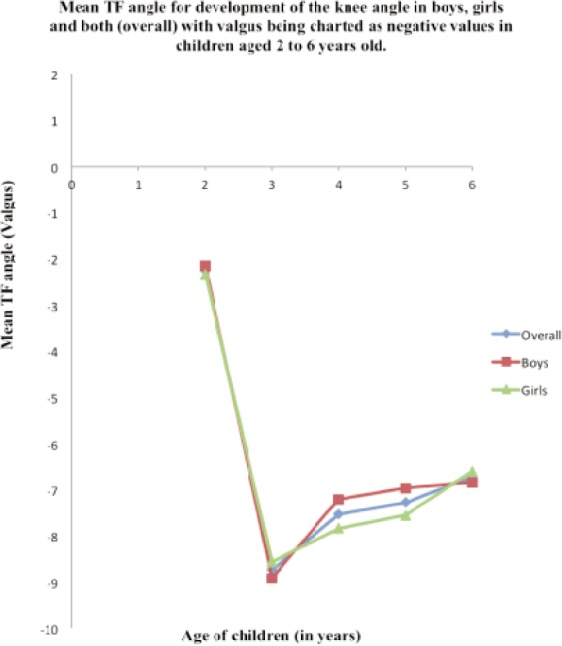
Line graph showing mean tibiofemoral angle for development of the knee angle in boys, girls and both (overall) with valgus being charted as negative values in children aged 2 to 6 years old.

There were 160 children (320 limbs) involved in this study. The mean of tibiofemoral angle for children at the age groups of 2 years, 3 years, 4 years, 5 years and 6 years were 2.25^o^ (SD= 0.53), 8.73^o^ (SD= 0.95), 7.53^o^ (SD= 1.40), 7.27^o^ (SD= 1.14) and 6.72^o^ (SD= 0.98) respectively (Table I). The pattern of tibiofemoral angle showed a steep increase of tibiofemoral angle from 2 years old to the maximum angle at 3 years old and gradually decrease from 3 to 5 years old (Fig. 1). The maximum mean (SD) tibiofemoral angle at 3 years old for boys, girls and all children were 8.91^o^ (SD=1.17), 8.56^o^ (SD=0.62) and 8.73^o^ (SD= 0.95) respectively. Independent t-test showed no significant difference of mean tibiofemoral angle between boys and girls except for those at the age group of 5 years (Table II).

**Table 5.1:** Mean (SD) tibiofemoral angle and confidence intervals distribution (95% CI) at different age and gender (n=160).

Age (in years)	No of limbs	Both boys & girls Mean (SD) of TFA	95% CI of TFA	No of limbs	Boys Mean (SD) of TFA	95% CI of TFA	No of limbs	Girls Mean (SD) of TFA	95% CI of TFA
2	64	2.25 (0.53)	(2.12, 2.38)	32	2.16 (0.51)	(1.97, 2.34)	32	2.34 (0.55)	(2.15, 2.54)
3	64	8.73 (0.95)	(8.50, 8.97)	32	8.91 (1.17)	(8.48, 9.33)	32	8.56 (0.62)	(8.34, 8.79)
4	64	7.53 (1.40)	(7.18, 7.88)	32	7.22 (1.41)	(6.71, 7.73)	32	7.84 (1.35)	(7.46, 8.33)
5	64	7.27 (1.14)	(6.98, 7.55)	32	6.97 (1.26)	(6.52, 7.42)	32	7.56 (0.95)	(7.22, 7.90)
6	64	6.72 (0.98)	(6.47, 6.96)	32	6.84 (0.99)	(6.49, 7.20)	32	6.59 (0.98)	(6.24, 6.95)

**Table 5.2:** Comparison of mean difference of tibiofemoral angle between boys and girls from age group 2 to age 6 group (n=160).

Age (in years)	Boys (n=80) Mean (SD)	Girls (n=80) Mean (SD)	Independent t- test Mean diff (95% Ch)	t-statistic (df)	p value
2	2.16	2.34	-0.19	-1.41	0.162
	(0.51)	(0.55)	(-0.45, 0.08)	(62)	
3	8.91	8.56	0.34	1.47	0.148
	(1.17)	(0.62)	(-0.13, 0.81)	(62)	
4	7.22	7.84	-0.63	-1.82	0.074
	(1.41)	(1.35)	(-1.31, 0.06)	(62)	
5	6.97	7.56	-0.59	-2.13	0.037
	(1.26)	(0.95)	(-1.15, -0.04)	(58)	
6	6.84	6.59	0.25	1.02	0.313
	(0.99)	(0.98)	(-0.24, 0.74)	(62)	

## Discussion

The value of normal physiological range of the tibiofemoral angle in specific population would help clinicians to decide on further treatment plan without having to expose children to unnecessary radiations, orthotics or braces.

Clinical measurement of tibiofemoral angle had been shown to have good correlation with roentgenographic measurement^1^. Many investigators chose the clinical method of measurement with goniometer because it was radiation-free, cheap and easily performed^2,3,7^. Mathew *et al*^7^ had found the clinical measurement of the tibiofemoral angle to have minimal intra-observer variability and high inter-observer reliability. In this study we also found a high inter-observer reliability by with intra-class correlation coefficient (ICC) of 0.87. By having an equal large sample of 64 for each age group and 32 for each gender subgroup, this study would be reliable to give conclusion on the timing of maximum valgus angle occurrence and differences between genders in the study group.

We focused on children between age group of 2 to 6 years because most of the changes occur between these age groups. The age group of 2 years includes children from 18 months and all of them were able to walk. Before walking age, most children have physiological genu varus^8^. Salenius and Vankka^1^ postulated that when a child starts to learn how to walk, he/she would increase the stability by holding the feet wide apart. This posture will increase the pressure over the lateral part of epiphyseal growth plate resulting in a slower growth process as compared to the medial part of epiphyseal growth plate, thus leading to genu valgus. Oyewole *et al*^9^ found that varus shape tibiofemoral angle decreased abruptly in the first 3 months of life and decreased slowly after that until it turned to valgus. Unfortunately in our study we did not include timing when the children started to walk.

The presence of valgus alignment of tibiofemoral angle in Malay from age of 2 to 6 years, as found in this study, is similar to Finland, Chinese, American, Turkish, Nigeria, Korean, Indian and Saudi children^1,2,3,4,6,10,11,12^.

Our study showed that there was no significant difference in the development of the tibiofemoral angle between boys and girls between 2 to 6 years old except for 5 years old group. Many other studies reported no tibiofemoral angle difference between genders in children between 2 to 6 years old^1,2,3,13^. There were two other studies reported a statistically significant differences between the gender on children between 5 to 6 years old^4,6^. Similar to our study, Rahman *et al*^6^ found difference of tibiofemoral angle of less than one degree with more valgus in girls. Reason for differences to occur at this age group remained to be explored.

In our study, we found that the age when maximum valgus angle achieved in Malay children was 3 years old. The children from Finland, China, America, Nigeria, Korea, and Saudi Arabia also achieved maximum valgus angle between 3 to 4 years old^1,2,6,10,11,12^. In contrast, maximum tibiofemoral angle was shown to occur at 6 years old in female and 7 years old in male Turkish children^3^. In Indian children, it occurred at 5 years old in boys and 6 years old in girls^4,7^.

The mean maximum valgus angle of 8.7^0^ (SD=0.95) in this study was comparable with Chinese, Turkish, American, Korean, Indian and Saudi Arabian children^2,3,4,6,10,12^. A slightly lower maximum valgus angle was found in Nigerian children (7.1^0^) and South Indian children (6.7^0^ to 7.3^0^)^7,11^. We did not compare our finding with the study by Salenius and Vankka^1^ who showed higher maximum valgus angle of 12^0^ as they measured the tibiofemoral angle by using radiograph.

## Conclusion

This study had shown that measurement of TF angle using the clinical method had a very good inter-observer reliability. The pattern of tibiofemoral angle in Malay children population between 2 years old to 6 years old is comparable to children elsewhere. The TF angle in Malay population was valgus since age of 2 years with the maximum angle of 8.73^0^(SD=0.95) achieved at age of 3 years. The TF angle in boys and girls only had no significant difference except at age of 5 years.
